# HMGCR: a malignancy hub - frontiers in cancer diagnosis and therapy

**DOI:** 10.3389/fonc.2025.1698320

**Published:** 2025-12-10

**Authors:** Yisong Yang, Yiting Liu, Teng Zou, Jiazhuo Liu, Xingzhi Zhou, Ran Tao, Shuangping Liu

**Affiliations:** 1 Engineering Technology Research Center for Functional Component Utilization of Organic Natural Products, Medical College, Dalian University, Dalian, Liaoning, China; 2 Department of Anatomy, Medical College, Dalian University, Dalian, Liaoning, China; 3 Dalian Institute of Marine Traditional Chinese Medicine, Dalian, Liaoning, China

**Keywords:** cholesterol metabolism, HMGCR, tumor microenvironment, signal pathways, TNF-α

## Abstract

Cholesterol metabolism is significantly activated during most tumor growth. As a key rate-limiting enzyme in cholesterol synthesis, HMG-CoA reductase (HMGCR) affects tumor metabolic reprogramming by upregulating cholesterol metabolism and promotes tumor growth and immune escape by remodeling the tumor microenvironment. It fuels tumor growth by providing cholesterol and isoprenoids, regulating critical pathways (Hippo, Hedgehog, MAPK), and modulating ferroptosis sensitivity. A complex bidirectional relationship exists between HMGCR and the pro-inflammatory cytokine TNF-α: TNF-α can inhibit HMGCR activity and thus inhibit cholesterol synthesis. At the same time, HMGCR influences TNF-αmediated inflammation and immune evasion. Statins, as HMGCR inhibitors, have shown anti-tumor effects in experiments. However, clinical application faces challenges including highly toxic concentration, drug resistance and tissue specificity. Accordingly, further exploration of mechanism-based targeted precision therapies to intervene in the HMGCR-TNF-α axis and related pathways, as well as the development of novel HMGCR inhibitors or optimization of existing drugs, represents an innovative strategy to enhance cancer treatment efficacy and advance drug development.

## 1 Introduction

Demographic predictions predict that new cancer cases may reach 35 million annually after 2050, representing a 77% increase compared to 2022 (1). Lung cancer and breast cancer are the most prevalent cancers among men and women respectively worldwide, and both are the leading causes of cancer-related deaths. Cancer screening, early diagnosis, and treatment are primary clinical research focuses and key strategies to reduce cancer mortality (2). The homeostasis of lipid metabolism is crucial for maintaining normal physiological functions in the human body. Its dysregulation may lead to cellular functional failure, immune imbalance, and multisystem diseases, thereby increasing the risk of cancer. Consequently, regulating lipid metabolic imbalances has become an important therapeutic strategy for tumors and multisystem diseases, etc. Studies have shown that cholesterol metabolism is upregulated during most tumor growth, and its metabolites may be indirectly involved in energy supply in lipid metabolism (3). Significant activation of cholesterol can regulate the tumor microenvironment, promote tumor progression, and influence the function of immune cells (4). This regulation alters the metabolic state of immune cells, inducing the activation and dysfunction of immunosuppressive cells, and enabling tumors to evade immune surveillance (5). Therefore, inhibiting cholesterol metabolism is one of the methods of tumor treatment.

Tumor necrosis factor-alpha (TNF-α) is a pro-inflammatory cytokine that can act as both a promoter and an inhibitor in tumors (6), impacting tumor progression. 3-Hydroxy-3-methylglutaryl coenzyme A reductase(HMGCR) is the first rate-limiting enzyme in cholesterol synthesis. Its expression level directly affects the rate of cholesterol synthesis. HMGCR also participates in cholesterol transport and storage, playing a critical role. Various factors regulate the activity of this enzyme. Notably, the interaction between TNF-α and HMGCR is significant in several diseases, providing new theoretical bases and potential therapeutic targets for tumor treatment.

## 2 Biological characteristics of HMGCR

### 2.1 Physiological role of HMGCR

HMGCR is an integral membrane protein composed of 888 amino acids in the endoplasmic reticulum. Its gene locus resides on chromosome region 5q13.3-5q14 (7). Human HMGCR has three subtypes, with subtype 1 playing a role in the progression of various diseases and being considered a potential therapeutic target (8). The expression and activity of HMGCR are regulated by multiple mechanisms, such as transcription, post-translational modifications, and negative feedback regulation (9). The expression of HMGCR is determined by sterol regulatory element-binding protein 2 (SREBF-2), a transcription factor that binds during the transcription process. HMGCR is the rate-limiting enzyme in cholesterol synthesis, and its post-translational modifications are crucial for maintaining the dynamic equilibrium of cholesterol production. When intracellular cholesterol or metabolic intermediates increase, HMGCR undergoes rapid degradation via the ubiquitin-proteasome system, preventing excessive cholesterol accumulation. This process represents the core negative feedback mechanism in cholesterol metabolism. This process is primarily mediated by E3 ligases such as gp78, RNF145, and TRC8, with the bridging action of Insig, via ubiquitinated lysine residues at Lys-89 and Lys-248 (10). When energy (ATP) is insufficient, AMPK is activated, leading to the deactivation of HMGCR through phosphorylation at Ser872, resulting in a rapid reduction in cholesterol synthesis (11). This regulatory network enables HMGCR to respond swiftly to cellular energy, nutritional, and metabolic signals. Defects in HMGCR ubiquitination and phosphorylation lead to cholesterol metabolism disorders, making them a potential target for drug development (12).

**Figure 1:**
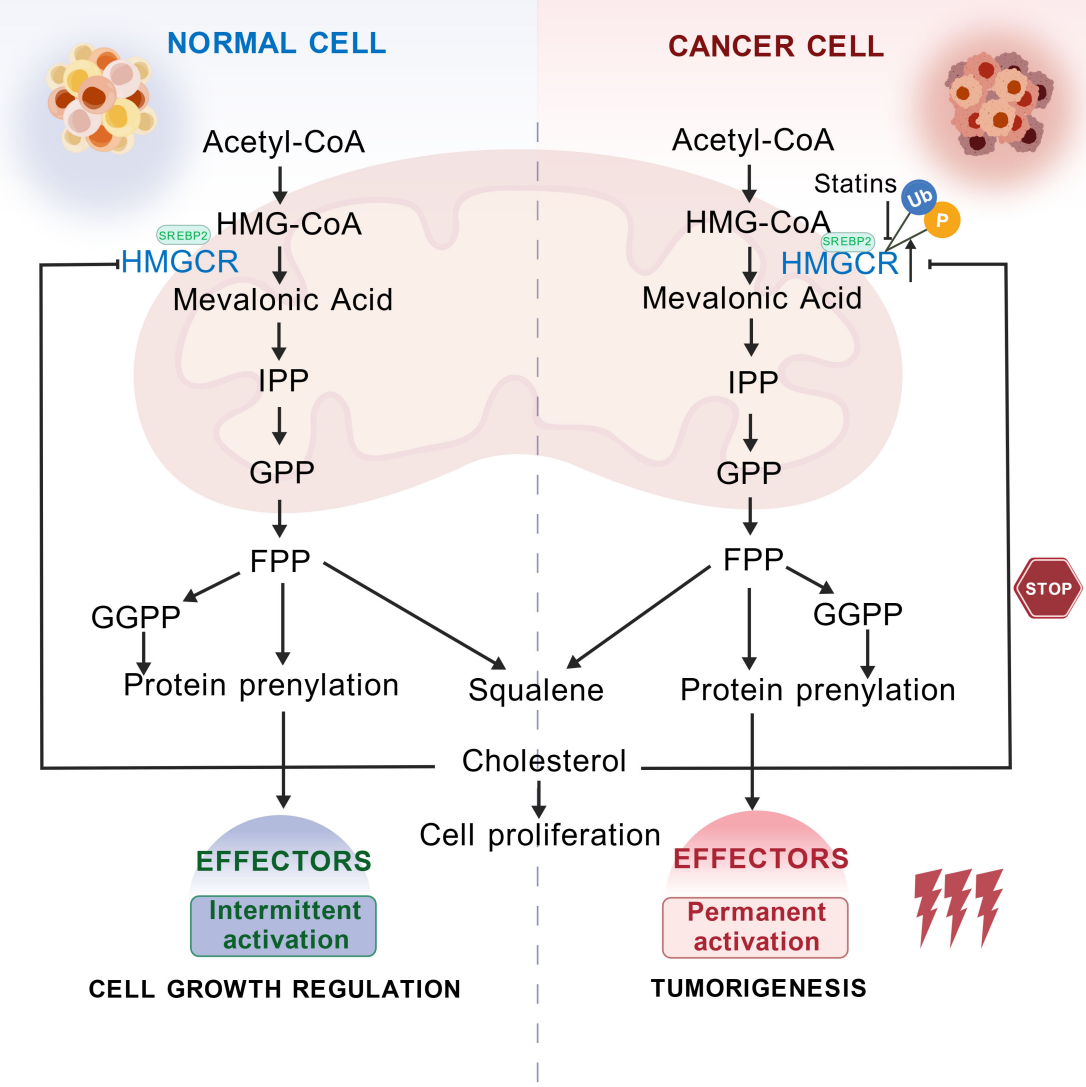
Schematic diagram of the physiological and pathological roles of HMGCR. HMGCR catalyzes the conversion of HMG-CoA to MVA. MVA is converted through a series of enzymatic reactions to form isoprenoid units, specifically isopentenyl pyrophosphate (IPP). These IPP units undergo further condensation to form squalene, which is ultimately converted into cholesterol, a molecule essential for life. Under physiological conditions, elevated intracellular cholesterol levels trigger a negative feedback mechanism that targets HMGCR, leading to its phosphorylation and subsequent ubiquitination, thereby reducing cholesterol synthesis and maintaining homeostasis. In pathological contexts, such as cancer, HMGCR is continuously expressed. This sustained expression drives persistent cholesterol synthesis, which, in turn, affects tumor progression. Inhibiting HMGCR expression represents a novel therapeutic approach in cancer treatment. Created with BioGDP.com (16).

The mevalonate pathway is closely related to cellular signal transduction and protein modification processes. It is also the core pathway for cholesterol synthesis, where intermediates are essential precursors for cholesterol production (13). Within this pathway, HMGCR catalyzes a two-step reduction of HMG-CoA to produce mevalonate, affecting the rate of endogenous cholesterol synthesis and influencing the prenylation of proteins involving isoprenoid compounds (14). Interestingly, HMGCR expression is tightly regulated by cholesterol and its derivatives. When there is a need to synthesize endogenous cholesterol, the expression of HMGCR is upregulated to promote cholesterol production. However, the excessive accumulation of intermediate metabolites, such as 25-hydroxycholesterol, sterols, or cholesterol, triggers strong, harmful feedback mechanisms that inhibit HMGCR expression. This regulation ensures the dynamic balance of cholesterol levels within cells (12, 15). Therefore, HMGCR plays a central role in maintaining cholesterol metabolic homeostasis, and its dysregulation is closely associated with various diseases. At the same time, HMGCR is a key target for statin medications in treating cardiovascular and cerebrovascular diseases. The roles of HMGCR in physiological and pathological contexts are summarized in **Figure 1**.

### 2.2 Effects of HMGCR in tumors

Disruption of cholesterol metabolism is a key factor driving tumor progression. Among the related pathways, the mevalonate (MVA) pathway, which serves as the core route for cholesterol synthesis, increases the risk of cancer when dysregulated and represents a potential target for anti-tumor therapy. Although this pathway has been extensively studied in cardiovascular diseases, its role in tumorigenesis requires further exploration. However, different cancer types exhibit varying degrees of dependence on cholesterol synthesis, uptake, and transport. Among them, prostate cancer, gastric cancer, colorectal cancer, and breast cancer show the most pronounced increase in endogenous cholesterol synthesis, with sustained activation of HMGCR and the transcription factor SREBP2 (17, 18). Therefore, HMGCR, as a candidate oncogene in these tumors, may represent a highly promising therapeutic target (19–21), offering new insights for interventions targeting cholesterol synthesis and its regulatory pathways (e.g., statins, SREBP inhibitors). As the key enzyme in cholesterol synthesis, HMGCR is required only to maintain basal cholesterol production under normal physiological conditions. When cholesterol levels become excessive, a corresponding negative feedback mechanism is triggered, inhibiting cholesterol synthesis to maintain cellular cholesterol homeostasis (12, 15). Conversely, in pathological states (such as cancer), tumor cells undergo metabolic reprogramming to meet their substantial nutritional demands, leading to persistently increased expression of HMGCR (22). Therefore, the expression of HMGCR differs between tumor and normal tissues.

Further research has found that HMGCR expression varies significantly among different cancer types (8). In cancers such as lung cancer, gastric cancer, colorectal cancer, glioblastoma, and prostate cancer, HMGCR is often highly expressed, and this high expression correlates with poorer overall survival in patients (23–25). Functionally, HMGCR overexpression can upregulate tumor proliferation-related genes, including K-RAS and mTORC1 (26). However, HMGCR is not highly expressed in all types of cancer. For example, some breast cancer subtypes exhibit low HMGCR expression (27). This phenomenon may be linked to tumor type and tissue specificity, and is influenced by the status of hormone receptors, such as the estrogen receptor (ER) (28). In summary, the expression and role of HMGCR in cancer are highly complex and tissue-specific, and its significance must be analyzed in the context of specific cancer types, subtypes, and clinical backgrounds.

### 2.3 Complex interactions between HMGCR and the tumor microenvironment

The tumor microenvironment regulates HMGCR at both transcriptional and post-translational levels through multiple pathways (mesenchymal cell paracrine signaling, hypoxia, AMPK-mediated phosphorylation, inflammatory cytokines), thereby influencing cholesterol metabolism. For example, in prostate cancer, HMGCR may form an autocrine or paracrine positive feedback loop that alters tumor cell proliferation (29). Under hypoxic conditions, the accumulation of the transcription factor hypoxia-inducible factor 1 (HIF-1) increases HMGCR levels and activity, thereby elevating tumor cell HMGCR expression and enhancing cholesterol and isoprenoid metabolism to support cell proliferation and angiogenesis (30). During mammalian cholesterol synthesis, AMPK phosphorylation inhibits HMGCR expression, thereby blocking cholesterol biosynthesis and enhancing cellular sensitivity to statin drugs (11). Inflammatory cytokines (such as tumor-associated macrophages, IL-6, VEGF, and TNF-α) can indirectly elevate HMGCR expression or activity, thereby promoting cholesterol synthesis. These regulatory mechanisms play a crucial role in tumor growth, angiogenesis, and sensitivity to metabolic-targeted drugs (such as statins), providing a theoretical basis for further metabolic interventions.

Additionally, elevated HMGCR expression leads to cholesterol metabolic reprogramming and modulates immune cell function within the tumor microenvironment (TME). tumor cells frequently exhibit HMGCR upregulation, resulting in substantial intracellular accumulation of cholesterol and its metabolites (29). Excess cholesterol alters the structure of cellular lipid rafts, enhancing the activity of signaling pathways such as PI3K/Akt and YAP (31). This promotes the secretion of pro-inflammatory/immunosuppressive factors (IL-6, VEGF, GM-CSF). These factors drive myeloid precursor cells in the bone marrow to differentiate into MDSCs. High levels of MDSCs suppress the function of NK and CD8+ T cells, promoting tumor immune escape (32). Concurrently, elevated HMGCR expression in tumor tissues enhances cholesterol synthesis within TAMs, promoting M2 (immunosuppressive) polarization. These TAMs secrete immunosuppressive factors, such as IL-10 and TGF-β, which weaken the antitumor activity of CD8+ T cells while facilitating tumor angiogenesis and immune evasion (33). Moreover, HMGCR upregulates PD-1 expression in Tregs via the p38 MAPK/GSK3β axis, thereby enhancing Tregs’ suppression of antitumor immunity and supporting tumor progression (34). Thus, HMGCR serves not only as a key metabolic node in tumor cells but also as a crucial regulator of immune cell function within the tumor microenvironment.

TNF-α is one of the key factors closely associated with cholesterol metabolism and the tumor microenvironment. As a core pro-inflammatory cytokine, TNF-α plays a vital role in chronic inflammatory responses and tumorigenesis. In chronic immune disease models, a regulatory relationship has been observed between TNF-α and HMGCR. For instance, TNF-α stimulation suppresses miR-146b-3p, leading to elevated HMGCR protein levels and thereby enhancing TNF-α-induced macrophage migration, invasion, and inflammatory responses (35). This finding suggests that TNF-α can directly increase HMGCR expression by modulating the microRNA pathway. Furthermore, studies have shown that HMGCR expression levels positively correlate with TNF-α levels (36), suggesting that HMGCR and TNF-α may influence related pathways and inflammatory responses by regulating lipid metabolism, thereby forming a mutually reinforcing cycle. Thus, investigating how TNF-α and HMGCR regulate the tumor microenvironment and downstream inflammatory reactions is crucial for understanding tumor progression mechanisms and identifying novel therapeutic targets.

## 3 Relevant pathways regulating HMGCR affecting tumor development

### 3.1 HMGCR inhibits ferroptosis in tumor cells

Ferroptosis is a newly identified form of programmed cell death. Since ferroptosis can effectively kill tumor cells, inducing ferroptosis in tumor cells has become a novel strategy for cancer therapy. Some researchers have pointed out that dysregulation of lipid metabolism affects cellular sensitivity to ferroptosis (37) and may also directly influence the initiation and progression of ferroptosis by regulating lipid synthesis and metabolic pathways (38). Multiple nodes in the cholesterol synthesis pathway (such as the mevalonate pathway) are crucial for regulating ferroptosis. For example, intermediate products in the cholesterol synthesis pathway, such as IPP, squalene, and coenzyme Q10, have a significant impact on ferroptosis regulation. Among these, IPP directly influences the occurrence and susceptibility to ferroptosis: it is not only a key component required for the maturation of selenocysteine tRNA but also participates in the synthesis of active glutathione peroxidase 4 (GPX4), an essential component of the antioxidant system (39). Therefore, the cholesterol synthesis pathway may provide new therapeutic targets for ferroptosis-related diseases.

Studies on hepatocellular carcinoma have revealed that activation of the oncogene BRCC36 promotes HMGCR deubiquitination, leading to its translocation from the plasma membrane to the endoplasmic reticulum (40). This translocation inhibits ferroptosis while promoting necrotic apoptosis. Conversely, BRCC36 inhibition reduces HMGCR stability, thereby activating ferroptosis and suppressing necrotic apoptosis. Notably, HMGCR also forms complex regulatory networks with other ferroptosis-related proteins such as GPX4, SLC7A11, and Nrf2. For instance, HMGCR enhances GPX4 and coenzyme Q synthesis, thereby inhibiting cellular ferroptosis (40). Studies indicate that HMGCR acts as a negative regulator of ferroptosis; knocking out HMGCR promotes ferroptosis in tumor cells (41). Concurrently, iron (Fe²^+^) is an essential driver of ferroptosis, with iron uptake, storage, efflux, and transcriptional regulation collectively influencing iron homeostasis. The interplay between these factors determines the sensitivity of tumor cells to ferroptosis. Thus, enhancing iron uptake, reducing iron storage and efflux, and decreasing HMGCR expression in tumors offer novel therapeutic directions. Furthermore, within the inflammatory microenvironment, the concentration and duration of TNF-α determine whether it promotes or inhibits ferroptosis. TNF-α enhances lipid peroxidation and ferroptosis by downregulating GPX4 expression, thereby weakening cellular antioxidant capacity and activating lipid reactive oxygen species (ROS) production through the NF-κB signaling pathway (42). However, in colorectal cancer, TNF-α induces ILF3 expression, which stabilizes SLC3A2 mRNA, enhances cystine uptake and GSH synthesis, ultimately reducing cancer cell susceptibility to ferroptosis (43). Thus, TNF-α exhibits a “double-edged sword” effect in the cancer microenvironment. Given the pivotal roles of HMGCR and TNF-α in regulating ferroptosis, complex interactions between these two molecules within the ferroptosis pathway are hypothesized to exist. Under specific tumor microenvironments (e.g., chronic inflammation, persistently elevated TNF-α levels), HMGCR and TNF-α may form intricate regulatory networks that either antagonize or synergistically promote inflammatory cell death. The specific outcome depends on tumor type, signaling intensity, and the expression status of other ferroptosis-related factors (e.g., GPX4, SLC7A11, Nrf2). Deepening our understanding of the molecular mechanisms underlying the HMGCR-TNF-α axis in regulating ferroptosis, in conjunction with tumor type, TNF-α signaling intensity, and HMGCR expression levels, may provide novel therapeutic targets for cancer treatment.

### 3.2 HMGCR inhibits apoptosis in tumor cells through the P38 MAPK pathway

The mitogen-activated protein kinase p38 (p38 MAPK) pathway constitutes a cascade of reactions that play a critical role in cellular signal transduction and metabolic regulation. This pathway influences cell apoptosis and inflammatory responses by regulating downstream effector molecules, such as TNF-α, interleukin-1 (IL-1), and the NF-κB signaling pathway (44). Research has demonstrated that lipid metabolism can modulate the MAPK pathway, as a reduction in cholesterol synthesis has been shown to activate the p38 MAPK pathway, resulting in cell apoptosis (45).

Although no direct interaction between HMGCR and p38 MAPK has been reported, they may exhibit synergistic effects during specific pathological processes. For example, a study on anti-tumor immunity found that activating AMP-activated protein kinase (AMPK) inhibits HMGCR expression, which subsequently activates the p38 MAPK pathway. This activation promotes the phosphorylation of GSK3β, which suppresses the expression of programmed cell death protein 1 (PD-1), thereby enhancing the anti-tumor activity of T cells (34). Additionally, knocking down HMGCR has been shown to promote p38 MAPK-mediated apoptosis and autophagy, effectively exerting anti-tumor effects (46). HMGCR regulates the synthesis of cholesterol and isoprenoid precursors, which are essential substrates for the isoprenylation of small GTPases (Ras, Rho, Rac). Isoprenylation determines the membrane localization and activation of these GTPases, thereby regulating the cascade activation of p38 MAPK and influencing cell proliferation, migration, and survival (47). These findings reveal the potential roles of HMGCR and p38 MAPK in regulating tumor immunity. Moreover, the relationship between p38 MAPK and TNF-α is also complex. In some instances, p38 MAPK cooperates with TNF-α to induce apoptosis in tumor cells. For example, TNF-α rapidly phosphorylates p38 MAPK via the TAK1-MKK3/6 cascade, inducing apoptosis in glioblastoma cells (48). However, p38 MAPK activation in prostate cancer protects cancer cells from TNF-α-induced apoptosis (49). This illustrates the dual and tissue-dependent functions of p38 MAPK in cancer (50). Such complexity in tumor progression may be associated with specific mechanisms or proteins. In summary, although there is currently no experimental evidence demonstrating a direct interaction among HMGCR, p38 MAPK, and TNF-α, these molecules may indirectly influence tumor progression through shared or intertwined signaling networks (inhibiting HMGCR expression, activating the p38 MAPK pathway, enhancing TNF-α-mediated apoptosis or immune activation). Future studies may further investigate the interactions among these three molecules across various tumor types and their potential therapeutic applications in cancer treatment.

### 3.3 HMGCR exerts tumor inhibition through the Hippo pathway

The Hippo pathway primarily regulates cell proliferation (51) and is a major tumor suppressor pathway. However, this pathway is frequently dysregulated in various cancers, including breast cancer, colorectal cancer, gastric cancer, liver cancer, and lung cancer. Dysregulation causes its downstream effectors, YAP/TAZ, to become hyperactivated. Hyperactivated YAP/TAZ translocate into the nucleus, promoting cell proliferation, epithelial-mesenchymal transition (EMT), tumor metastasis, and the acquisition of stem-like properties (52, 53).

HMGCR serves as the rate-limiting enzyme of the MVA pathway, catalyzes the conversion of HMG-CoA to mevalonate, and provides precursors for the synthesis of isoprenoids such as geranylgeranyl pyrophosphate (GGPP). These isoprenoids are essential for the prenylation, membrane association, and kinase activation of Rho GTPases (54). Rho GTPases play a central role in the Hippo signaling pathway by regulating the dynamics of the cytoskeleton, which in turn modulates the activity of YAP/TAZ (53). Therefore, inhibiting HMGCR (e.g., with statins) reduces GGPP production, weakens Rho GTPase activity, and restores Hippo core kinase activity (MST1/2-LATS1/2). This inhibits YAP/TAZ nuclear translocation and transcriptional activity, ultimately suppressing the proliferation and metastasis of cancer cells (3, 55). This mechanism explains how small-molecule inhibitors, such as statins, can inhibit YAP/TAZ nuclear localization and transcriptional functions (3), revealing a positive feedback regulatory loop among lipid metabolism (HMGCR), cytoskeleton dynamics (Rho), and the Hippo pathway (YAP/TAZ). Moreover, the Hippo signaling pathway and the TNF-α signaling pathway also demonstrate synergistic interactions. TNF-α enhances YAP/TAZ expression via NF-κB or directly inhibits LATS activity, thereby increasing YAP/TAZ nuclear localization and promoting inflammation-driven cancer cell invasion and metastasis. For example, in breast cancer, these two pathways enhance YAP transcriptional activity, promoting the expression of hexokinase 2 (HK2), which in turn increases cancer cell migration and invasion (56). It remains unclear whether TNF-α plays a role in the effect of HMGCR on the Hippo signaling pathway. However, HMGCR and TNF-α co-regulate the Hippo-YAP/TAZ signaling network through distinct yet intertwined mechanisms. HMGCR activates Rho-GTPases through its metabolites to inhibit core Hippo kinases, while TNF-α upregulates YAP/TAZ via NF-κB and suppresses LATS. Their synergistic action forms a metabolic-inflammatory-Hippo-positive feedback loop that drives tumor cell proliferation and metastasis. Multi-point intervention targeting this network (via statins, NF-κB inhibitors, and YAP/TAZ-TEAD inhibitors) offers a potential combination strategy for cancer therapy. Deepening our understanding of the specific mechanisms and interactions among these three factors across different cancer types not only provides new perspectives for tumor biology research but also lays a solid theoretical foundation for developing combination therapies targeting this network.

### 3.4 HMGCR affects the Hedgehog pathway to induce tumor cell proliferation stably

The Hedgehog (Hh) signaling pathway regulates cell proliferation, differentiation, and migration, and its dysregulation has been shown to promote tumor growth and metastasis (25). Notably, aberrant activation of Hh signaling is closely associated with the development of various malignancies, including basal cell carcinoma, ovarian cancer, gastric cancer, lung cancer, pancreatic cancer, and breast cancer (57).

As the rate-limiting enzyme of the MVA pathway, HMGCR indirectly influences the activation of the Hh signaling pathway by regulating cholesterol synthesis, thereby promoting tumor progression, and metastasis. Two main hypotheses have been proposed to explain how HMGCR modulates the Hh pathway (58): first, HMGCR may directly interact with Hh ligands, thereby upregulating the activity of Smoothened (Smo); second, HMGCR may indirectly exert effects by synthesizing lipid precursors, such as cholesterol or isoprenoid compounds, which provide a necessary lipid environment for Hh signal transduction. For example, in hepatocellular carcinoma, HMGCR expression levels in the Hh pathway were found to correlate positively with the receptor Smo. Cholesterol or isoprenoids synthesized by HMGCR promote SMO activation. The activated HH pathway upregulates the secretion of inflammatory mediators (such as IL-6 and TNF-α), thereby activating the downstream NF-κB signaling pathway and further enhancing Hh pathway activation. This cascade stimulates the maintenance of cancer stem cell (CSC) properties and metastatic capacity (25). Furthermore, GLI1 overexpression is recognized as a hallmark of HH pathway activation. HMGCR enhances HH signaling activity by promoting nuclear translocation of the downstream transcription factor Gli1 (25). Significantly, TNF-α may induce drug resistance in tumor cells by activating the Hedgehog (Hh) signaling pathway (59). In pancreatic cancer, TNF-α activates NF-κB, induces nuclear translocation of Gli1/GLI2, and enhances their transcriptional activity. This activates the non-canonical HH pathway, thereby promoting migration, invasion, and EMT in pancreatic ductal adenocarcinoma cells (60). In breast cancer, TNF-α induces Gli1 nuclear translocation, elevates MMP-9 expression, and drives cell invasion to confer resistance (61). HH signaling is critical for self-renewal in multiple tumor stem cells (gastric cancer, HCC, pancreatic cancer). Thus, HMGCR promotes classical SMO-Gli activation via metabolites, while TNF-αprimarily regulates Gli expression through NF-κB-mediated non-classical pathways or synergistically with other cytokines (e.g., TGF-β). It is hypothesized that in cells with high HMGCR expression, active cholesterol or isoprenoid metabolism facilitates SMO activation and signal transduction. Concurrently, TNF-α upregulates Gli transcription factor expression through pathways like NF-κB. Both pathways synergistically promote Hedgehog-associated phenotypes, such as cell proliferation and stemness maintenance, forming a positive feedback loop that drives cancer cell proliferation, migration, and immune microenvironment remodeling, thereby accelerating tumor progression. Therefore, a combined intervention targeting the complex regulatory network formed by HMGCR, TNF-α, and Hedgehog signaling pathways may offer novel therapeutic approaches to overcome tumor resistance and metastasis, thereby opening new avenues and strategies for cancer treatment.

### 3.5 Inhibition of HMGCR by TNF-α suppresses tumor development via cholesterol synthesis

Cholesterol regulates lipid metabolism and is essential for maintaining cellular homeostasis and normal physiological functions. However, abnormalities in cholesterol synthesis or metabolism can lead to excessive accumulation in the blood and tissues, thereby increasing the risk of cardiovascular diseases and other metabolic disorders (62, 63). At the same time, dysregulation of cholesterol metabolism has been confirmed as a critical driver of tumor development. Cancer cells enhance their ability to uptake cholesterol and significantly upregulate endogenous cholesterol synthesis. This phenomenon is associated with the overexpression of key enzymes, such as HMGCR, and transcription factors, including SREBP, involved in the cholesterol biosynthesis pathway (62, 64). Given the apparent association between abnormal cholesterol metabolism and cancer, targeting the cholesterol biosynthesis pathway has become a hotspot in cancer therapeutics.

**Figure 2:**
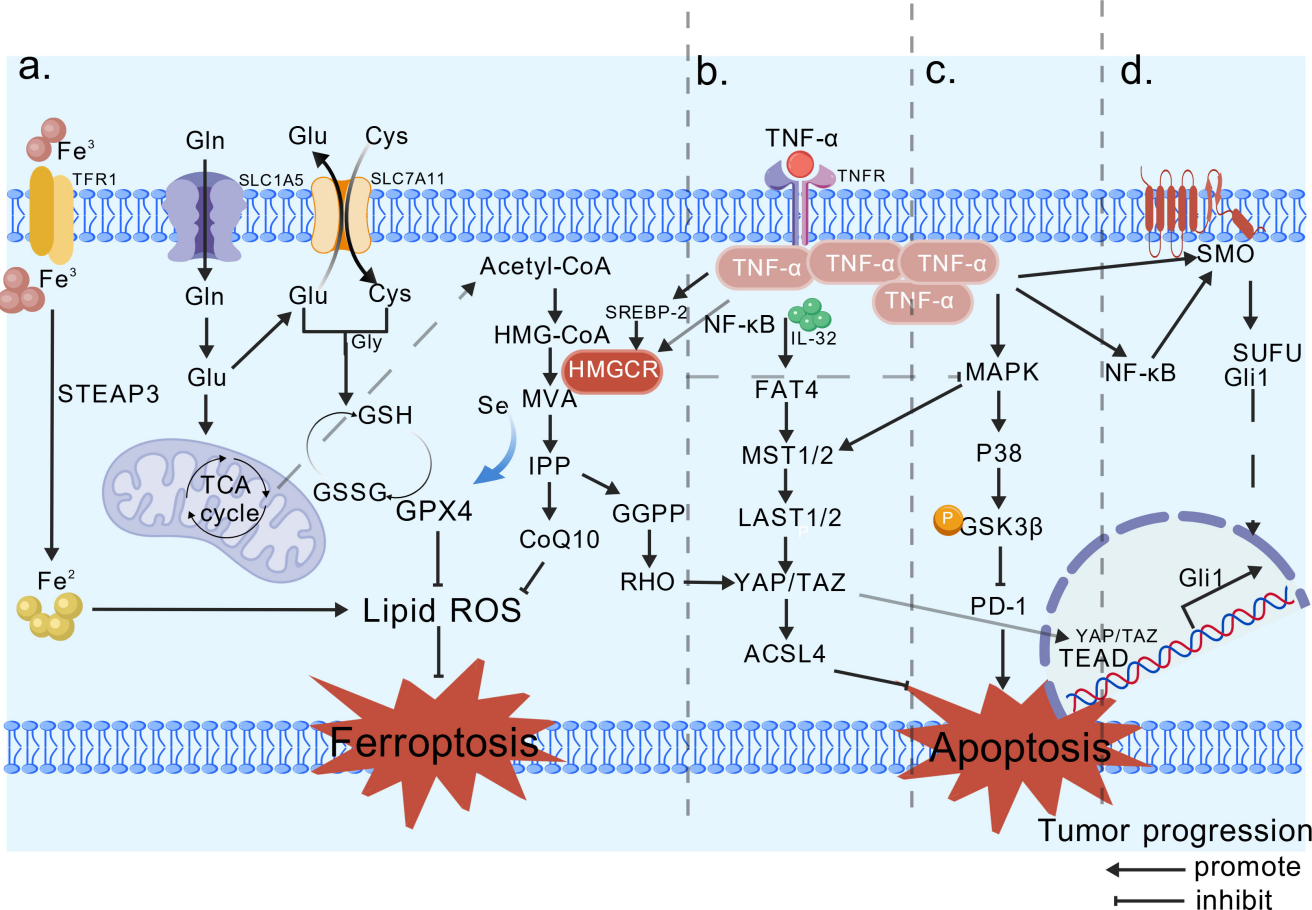
Relevant pathways involved in tumor development by HMGCR. **(A)** Ferroptosis pathway, HMG-CoA is converted into MVA under HMGCR, and IPP binds to synthesize GPX4, thereby inhibiting ferroptosis. **(B)** Hippo pathway, GGPP promotes the nuclear translocation of YAP/TAZ, driving cancer progression. TNF-α upregulates YAP/TAZ expression via NF-κB, thereby enhancing YAP’s transcriptional activity. **(C)** The P38-MAPK pathway, HMGCR indirectly inhibits, promoting PD-1 and suppressing tumor apoptosis. TNF-α rapidly phosphorylates p38 MAPK via the MKK3/6 cascade. **(D)** Hedgehog, HMGCR is positively correlated with the receptor Smo. TNF-α activates NF-κB, inducing the nuclear translocation of Gli1/GLI2, thereby suppressing tumor cell apoptosis via the Hedgehog signaling pathway. It is hypothesized that TNF-α may enhance the transcription and activity of HMGCR through the NF-κB/SREBP-2 pathway, thereby supporting tumor cell proliferation and migration. Created with BioGDP.com (16).

Intermediate metabolites of the cholesterol synthesis pathway, including mevalonate, isoprenoids, and sterols (65), closely relate to the progression of cancer. When cancer cells sense a decrease in cholesterol levels, they activate the PTEN/AKT/mTORC1 signaling axis, which promotes the expression of SREBF-2 (66). Subsequently, the SREBF-2 activates the transcription of HMGCR (67), thereby increasing cholesterol content. In most tumors, the activity and expression of HMGCR are enhanced, thereby increasing the supply of key downstream metabolites that modulate lipid metabolism to promote cell proliferation and transformation, ultimately accelerating cancer progression (68). Notably, TNF-α plays a key inhibitory role in regulating cholesterol synthesis. Human studies have demonstrated that TNF-α suppresses cholesterol synthesis through multiple mechanisms, with HMGCR being one of its primary targets (69). TNF-α activates the NF-κB signaling pathway, which induces the ubiquitination of HMGCR and accelerates its degradation, leading to decreased HMGCR protein levels and reduced enzymatic activity. Moreover, TNF-α can indirectly regulate HMGCR gene transcription by affecting the activity or stability of SREBP-2 (70). At the same time, TNF-α may influence HMGCR activity indirectly by modulating other cholesterol metabolism pathways, such as CYP7A1 and LDL receptor pathways (69). It is hypothesized that HMGCR and TNF-α jointly drive a tumor-growth-promoting microenvironment rich in cholesterol and inflammatory signals. Within this tumor microenvironment, cancer cells may develop resistance to the inhibitory effects of TNF-α. This resistance may be achieved by promoting TNF-α secretion to sustain high activity of the SREBP-HMGCR axis. Consequently, elevated HMGCR levels continuously synthesize cholesterol, while the inflammatory environment created by TNF-α may accelerate tumor progression. Given the complex interactions between TNF-α and HMGCR, elucidating their pathological mechanisms in diseases such as cancer and developing targeted intervention strategies is of significant importance. The related pathways of HMGCR in tumor progression are summarized in **Figure 2**.

## 4 Impact of HMGCR on tumor therapy

### 4.1 HMGCR inhibitors and diseases

Research on HMGCR is intensifying due to its key role in the mevalonate pathway. HMG-CoA reductase inhibitors (statins) are clinically used as cholesterol-lowering drugs and exhibit anti-inflammatory properties. Some studies have found that statins exhibit multiple anti-tumor effects, including inducing cell apoptosis and autophagy, inhibiting cell migration and invasion, and modulating pro-tumor signaling molecules and the immune system (counteracting cisplatin resistance) (71). Although patients using statins have a lower probability of developing cancer (72) and a reduced cancer mortality rate, this conclusion requires further confirmation through clinical trials involving cancer patients who are taking statins. Additionally, some studies and opinions suggest that there is a contradictory relationship between statins and cancer risk, and that statins may have no effect on cancer or even increase the risk of cancer (73, 74). Therefore, the use of statins in cancer treatment remains a complex and controversial issue.

Some studies suggest that statins may inhibit cholesterol synthesis in most tumors. This inhibition interferes with the metabolism and signaling pathways of tumor cells, thereby inhibiting their proliferation and migration. For instance, HMGCR inhibitors can suppress the expression of vascular endothelial growth factor (VEGF) and hinder tumor angiogenesis. Simvastatin inhibits the Hedgehog signaling pathway and reverses the properties of cancer stem cells (CSCs) in hepatocellular carcinoma (HCC), thereby suppressing tumor metastasis (44). Additionally, HMGCR inhibitors combined with radiotherapy can enhance the activation of the cGAS-STING pathway, promote the infiltration and function of CD8+ T cells, and improve anti-tumor efficacy (33). At the same time, statins may exert their antitumor effects by increasing oxidative stress (75). However, the anti-tumor efficacy of statins faces challenges in clinical application. Statins exhibit specific anti-tumor effects *in vitro* and animal experiments; however, the required drug concentrations are incredibly high and toxic, making it challenging to achieve such concentrations *in vivo* (76). Furthermore, statins may induce resistance during treatment. Long-term statin use can cause cancer cells to upregulate key enzymes such as HMGCR or GGPP synthase (GGPS1), enabling sufficient isoprenoid supply even when HMG-CoR is inhibited. Moreover, prolonged HMGCR inhibition by statins reduces intracellular cholesterol synthesis, causing the SCAP-SREBP-2 complex to dissociate from INSIG and translocate to the Golgi apparatus. This activates the intracellular SREBP-2 feedback loop, elevating HMGCR gene expression and partially counteracting statin suppression (77). Upregulation of the HMGCR/SREBP-2 axis has been identified as a key mechanism of resistance in tumor models, including those of prostate cancer and pancreatic cancer cells (78). CYP3A4, the primary metabolic enzyme for atorvastatin and simvastatin, exhibits genetic variations or drug interactions that can slow or accelerate the clearance of these drugs, thereby affecting the efficacy of statins. Inflammatory cytokines (IL-6, TNF-α) can enhance HMGCR activity and activate alternative synthases, enabling cholesterol synthesis via bypass pathways even when HMGCR is inhibited. This circumvents statin treatment efficacy. Concurrently, statin use reduces coenzyme Q10 synthesis, thereby limiting mitochondrial energy supply and triggering adverse muscle reactions, such as pain and fatigue (79). This complicates the determination of safe and effective anti-cancer dosages. Limitations in statin therapy, including insufficient clinical evidence, heterogeneous efficacy, pharmacokinetic constraints, drug resistance, potential adverse reactions, and drug interactions, restrict the application of monotherapy in tumor treatment. Therefore, a precision treatment regimen combining statins with other drugs is a viable option (80). Future research should focus on determining the optimal dosage and combination therapy strategies for HMGCR inhibitors across various tumor types to enhance treatment efficacy and minimize side effects. Additionally, the development of novel HMG-CoA reductase inhibitors to combat tumors should be explored. **Table 1** lists the HMGCR inhibitors, statins.

**Table 1:** Classification of statins in the function of lowering lipid levels.

Types	Pharmaceuticals	Pharmacokinetics	Metabolic pathways	Key transporters	Primary elimination pathways (liver/kidney)
I-Statins (Natural/ Semi-synthetic)	Lovastatin	Lipophilicity	CYP3A4 mechanism	OATP1B1, BCRP, MDR1	Liver extraction > 80%; Fecal excretion 58-70%
	Simvastatin	Lipophilicity	Not shown	OATP1B1, BCRP, MDR1	Liver extraction > 80%; Fecal excretion 58-70%
	Pravastatin (Not listed)	Hydrophilicity	Sulfation/Glucuronidation	OATP1B1	Renal excretion 20-60%
II-Statins (fully synthesis)	Atorvastatin	Lipophilicity	CYP3A4 mechanism	OATP1B1, BCRP	Hepatic clearance 70%; Renal clearance < 5%
	Pitavastatin	Lipophilicity	Glucuronidation	OATP1B1	Hepatic clearance 70%; Renal clearance < 5%
	Rosuvastatin	Hydrophilicity	CYP2C9 mechanism	OATP1B1, BCRP	Hepatic clearance 70%; Renal clearance < 5%
	Fluvastatin	Amphipathy	CYP2C9 mechanism	OATP1B1	Hepatic clearance 70%; Renal clearance < 5%

### 4.2 HMGCR and drug resistance and clinical prognostic value

Elevated cholesterol and isoprenoid compounds have been linked to drug resistance (81). Therefore, inhibiting their expression constitutes a key strategy to enhance drug sensitivity. Specifically, studies on glioblastoma have identified HMGCR expression and its mediated cholesterol synthesis as novel mechanisms of resistance (20). In lung cancer, increased exogenous cholesterol levels stimulate the expression of HMGCR, leading to resistance to cisplatin, a first-line anti-cancer drug. Conversely, knocking down HMGCR enhances drug sensitivity. Furthermore, HMGCR influences drug resistance by regulating the activity of P-glycoprotein (P-gp), encoded by the ABCB1 gene, which exhibits a multidrug resistance phenotype (82). These findings indicate that the degree of resistance is correlated with the expression of HMGCR in the cholesterol synthesis pathway. Notably, genetic polymorphisms of HMGCR itself can affect the cholesterol-lowering efficacy of statins (83, 84). For instance, in MCF-7 breast cancer cells, statin treatment induces the upregulation of SREBP-2 and HMGCR at both the gene and protein levels. The resulting high expression of HMGCR forms a feedback loop that contributes to statin resistance. Consequently, targeting the SREBP-2/HMGCR axis is an effective strategy to reverse statin resistance (85).

Given the established association of high cholesterol and sterol levels with increased cancer risk (81), it is imperative to investigate the significance of HMGCR expression in tumors. The expression of HMGCR correlates significantly with patient prognosis across multiple tumor types; however, such associations demonstrate tissue specificity, and the precise mechanisms and independent prognostic value remain to be elucidated. On one hand, in certain tumors, high HMGCR expression associates with poor prognosis and reduced survival. For example, HMGCR expression is significantly higher in hepatocellular carcinoma tissues than in adjacent noncancerous tissues, and its overexpression correlates with worse overall survival (OS) (20). Similarly, strong HMGCR expression in prostate cancer correlates with decreased biochemical recurrence (BCR)-free survival (86). On the other hand, in various tumor types, elevated HMGCR expression is associated with a better prognosis. This seemingly contradictory phenomenon may be related to the induction of apoptosis by isoprenoid substances generated in the mevalonate pathway (87, 88) or may be influenced by tumor-specific differences, such as estrogen receptor status. A study based on The Cancer Genome Atlas (TCGA) ovarian cancer dataset found that high HMGCR expression correlates with improved progression-free survival (PFS) (89). In colorectal cancer, HMGCR expression is associated with more advanced clinical staging but better prognosis (90). Taken together, HMGCR expression may serve as a potential prognostic marker, yet establishing its utility as an independent prognostic factor requires extensive experimental validation. Furthermore, the complex regulatory interactions between HMGCR expression and other genes and metabolic pathways may modulate its prognostic value; therefore, in-depth investigation into the specific roles and mechanisms of HMGCR in tumorigenesis and progression remains essential. **Table 2** summarizes the prognostic significance and drug resistance associated with HMGCR in tumors.

**Table 2:** The prognostic significance and drug resistance of HMGCR in tumors.

Tumor type	Expression	Prognosis	Antimicrobial resistance	Mechanism	Evidence hierarchy
Prostate cancer	High	Negative	Targeted therapy drugs	AR	I (MRI)/II (Tissue)
Glioblastoma	High	Negative	Targeted therapy drugs	Cholesterol metabolism	Not shown
Hepatocar cinoma	High	Negative	Statin	HMGCR compensation	Not shown
ER breast cancer	High	Negative	Statin	SREBP-2/HMGCR	II
Gastric cancer	High	Negative	Platinum	Cell membrane fluidity	III
Ovarian cancer	High	Positive	Platinum	LDLR, Cholesterol	I
Lung cancer	High	Positive	Platinum	P-gp	I
Colorectal cancer	High	Positive	Not shown	Not shown	I

## 5 Conclusion and prospects

In recent years, the role of cholesterol metabolism within the tumor immune microenvironment has attracted increasing attention. Inhibiting HMGCR may enhance the infiltration and function of CD8+ T cells, thereby improving the anti-tumor immune response (33). However, the mechanisms underlying the interaction between HMGCR and the immune system remain unclear. For example, whether the expression of HMGCR affects the immune evasion capability of tumor cells or whether its inhibition impacts the function of immune cells is a question that requires further investigation.

Given that HMGCR is highly expressed in most cancers and exhibits distinct prognostic significance across different cancer types, it can serve as a predictive marker and novel therapeutic target for specific tumors with markedly increased cholesterol synthesis, such as prostate cancer, gastric cancer, colorectal cancer, and breast cancer. Abnormal HMGCR expression in cancer represents both a hallmark of metabolic reprogramming and a driver of therapeutic resistance. HMGCR regulates cellular cholesterol content by stimulating cholesterol synthesis, thereby affecting membrane fluidity, receptor clustering, and signal transduction. The mevalonate pathway (initiated by HMGCR) promotes cholesterol, CoQ10, and IPP synthesis, upregulates membrane structure and steroid signaling, indirectly governs GPX4 antioxidant capacity, and blocks ferroptosis. In gastric cancer resistance models, upregulation of the cholesterol-HMGCR axis enhances lipid rafts and suppresses ferroptosis. Treatment with simvastatin (an HMGCR inhibitor) reverses resistance and restores ferroptotic effects (91). Thus, HMGCR occupies a central node at the crossroads of lipid synthesis, redox balance, and ferroptosis, representing a complex and multifaceted biological process in tumors. Moreover, HMGCR not only regulates ferroptosis but also serves as a crossroads hub for three key signaling networks: Hippo-YAP/TAZ, Hedgehog, and p38 MAPK (65). HMGCR comprehensively regulates cell proliferation, differentiation, and survival by controlling YAP/TAZ nuclear localization, enhancing Smo-GLI signaling, and inhibiting p38 MAPK phosphorylation, thereby synergistically promoting malignant tumor progression. Activation of the Hippo pathway reduces cellular stress and indirectly inhibits p38 MAPK; conversely, p38 MAPK activation modifies YAP/TAZ activity through phosphorylation. YAP/TAZ upregulates key Hedgehog genes (e.g., Gli1), while activated Hh signaling promotes YAP/TAZ nuclear localization and activates p38 MAPK via non-canonical pathways. Conversely, p38 modulates HH signaling intensity by directly phosphorylating GLI or regulating the expression of SMO and PTCH. HMGCR serves as a common node for all three pathways by simultaneously regulating YAP/TAZ, Smo/GLI, and p38 MAPK. Consequently, in tumors (e.g., prostate cancer, breast cancer), HMGCR overexpression establishes a dual Hippo-YAP/TAZ and Hedgehog pathway, while inhibiting p38 MAPK enhances cellular anti-apoptotic capacity. This convergence mechanism plays a central role in tumor progression, tissue regeneration, and drug-induced cellular injury across physiological and pathological contexts, providing a theoretical basis for combined therapies targeting HMGCR (e.g., statins) or its downstream pathways.

Notably, the interaction between HMGCR and TNF-α may synergistically promote tumor cell proliferation, invasion, and metastasis, while inhibiting apoptosis, thereby collectively driving tumor progression. In most tumor models, TNF-α acts primarily as an upstream factor, upregulating HMGCR transcription and activity through the NF-κB/SREBP-2 pathway. This provides cancer cells with cholesterol and isoprenylation precursors, supporting tumor cell proliferation and migration. HMGCR itself does not directly drive TNF-α production; however, inhibiting HMGCR (e.g., with statins) weakens lipid raft structures and small GTPase signaling, thereby indirectly reducing TNF-α secretion and inflammatory mediators via NF-κB. Based on existing evidence, it is reasonable to infer that high HMGCR expression amplifies TNF-α-driven EMT, angiogenesis, lymphangiogenesis, and p38 MAPK inhibition, thereby promoting metastatic dissemination. However, current literature indicates (92) that the interaction between HMGCR and TNF-α is not uniform across different tumor types and tumor immune microenvironments, but rather exhibits significant variability. In tumors with high endogenous HMGCR expression and abundant TNF-α (e.g., HER2^+^/TNBC breast cancer), statin-mediated HMGCR-TNF-α interactions are more pronounced, manifesting as: reduced HMGCR expression, suppressed TNF-α production, and consequently weakened pro-inflammatory/pro-angiogenic signaling. altering macrophage polarization (inhibiting M2 type, promoting M1 type), and enhancing CD8^+^ T cell infiltration. In tumors with low HMGCR expression and limited TNF-α (e.g., low-grade, hormone receptor-positive breast cancer), statins exhibit relatively weak regulatory effects on TNF-α, with overall interactions being less pronounced. In summary, the interaction between HMGCR and TNF-α exhibits varying intensity and biological consequences depending on tumor type (e.g., breast cancer subtypes, glioblastoma) and differences in the immune microenvironment (macrophage polarization, T cell infiltration levels). This suggests that a high HMGCR/high TNF-α phenotype may serve as a biomarker for sensitivity to combined HMGCR inhibitor and anti-TNF-α therapy, warranting further validation in future precision medicine trials. Future multi-level experimental approaches—including qRT-PCR, Western blot, Co-IP, immunofluorescence, and cholesterol synthesis tracing—can systematically validate whether TNF-α downregulates HMGCR expression and reduces cholesterol synthesis by inhibiting SREBP-2 processing and enhancing SCAP-INSIG binding.

Statins show promise in tumor treatment, but their limitations should not be overlooked. Although combining statins with other anti-tumor drugs may enhance anti-tumor effects, their effectiveness varies across different types of tumors. Meanwhile, the efficacy of combination therapy still needs further clinical trials for verification. Therefore, utilizing AI technology to assist in screening/designing targeted inhibitors, or improving existing ones, could be a promising strategy for drug development.

In conclusion, this review summarizes previous research findings and perspectives, detailing the regulatory mechanisms of HMGCR, including transcriptional regulation, post-translational modifications, and the involved signaling pathways, while also discussing the impact of TNF-α on tumors in conjunction with HMGCR. Since TNF-α and HMGCR exhibit complex interactions, further in-depth investigations into their mechanisms of action and mutual relationship will provide a more comprehensive theoretical foundation and therapeutic strategies for treating tumors and other diseases. Future research should concentrate on elucidating the specific mechanisms of HMGCR-TNF-α interactions across different diseases, determining whether combined therapies yield superior efficacy, and exploring potential interventions through epigenetic approaches. This will enable the development of novel treatments targeting this axis, paving the way for therapeutic breakthroughs.
